# Assessment of Electroacupuncture Therapy with Distant-Approximal Acupoints Based on the HPT Axis in Rats with Oligoasthenospermia Through Transcriptomic Analysis

**DOI:** 10.1007/s43032-025-01821-x

**Published:** 2025-02-27

**Authors:** Li Hongyu, Yang Nan, Li Kaiying, Zhao Zhenning, Zhao Lili, Mu Jing, Ma Huisheng

**Affiliations:** 1 Ningxia Medical University, Yinchuan, 750004 Ningxia Hui Autonomous Region China; 2 General Hospital of Ningxia Medical University, Yinchuan, 750004 Ningxia Hui Autonomous Region China; 3 Ningxia Medical University Key Laboratory of Ningxia Minority Medicine Modernization Ministry of Education, Yinchuan, 750004 Ningxia Hui Autonomous Region China; 4 Ningxia Regional Key Laboratory of Integrated Traditional Chinese and Western Medicine for Prevention and Treatment of Regional High Incidence Disease, Yinchuan, 750004 Ningxia Hui Autonomous Region China; 5 Ningxia Health Vocational and Technical College, Shizuishan, 753000 Ningxia Hui Autonomous Region China

**Keywords:** Oligoasthenospermia, Hypothalamic-pituitary–testicular axis, Transcriptome sequencing, Differentially expressed genes, Enrichment analysis

## Abstract

The transcriptomic analysis was used to explore the effect of electroacupuncture therapy with distant-approximal acupoints based on the hypothalamic-pituitary–testicular (HPT) on gene expression patterns and pathways in oligoasthenospermia (OAT) rats. In this study, the rat model of OAT after intragastric administration of adenine was selected as the research object, and randomly divided into a blank group (C), a model group (M), and a electroacupuncture therapy with distant-approximal acupoints group (D). After electroacupuncture intervention, the epididymal sperm quality and serum sex hormone levels of rats was detected and three tissue samples of HPT axis were taken, and differentially expressed genes (DEGs) were screened by transcriptome sequencing technology. GO functional annotation and KEGG pathway enrichment analysis were performed on the DEGs. The oxidative stress related indicators in serum and HPT axis were also detected to verify the transcriptomic analysis results. Compared with group C, group M rats showed a decrease in sperm count (*p* < 0.001), sperm survival rate (*p* < 0.001), and sperm motility rate (*p* < 0.001); the serum levels of GnRH in the group M rats decreased (*p* < 0.001), FSH increased (*p* < 0.001), LH increased (*p* < 0.001), and T decreased (*p* < 0.001). Compared with group M, group D rats showed an increase in sperm count (*p* < 0.01), sperm survival rate (*p* < 0.001), sperm motility rate (*p* < 0.001), an increase in GnRH levels (*p* < 0.001), a decrease in FSH levels (*p* < 0.01), a decrease in LH levels (*p* < 0.001), and an increase in T levels (*p* < 0.001). In bioinformatics analysis, compared with group M, we identified 1656, 518, 530 DEGs in the hypothalamus, pituitary, and testis in group D, respectively. Combining the go and KEGG analysis results, three oxidative stress signaling pathways that may be related to electroacupuncture intervention in OAT rats were screened. It mainly involves the glutamatergic synaptic pathway, the MAPK signaling pathway and the glutathione metabolism pathway. Six key genes (Gng12、Grin1、Gng7、Jun、Nf1 and Gstp1) were identified as key candidate genes regulating epididymal sperm quality on the HPT axis, which may affect the reproductive function of rats by affecting the process of oxidative stress in vivo. No matter in serum or in three tissues of HPT axis, GPX4 level in group M was decreased compared with Group K (*p* < 0.0001), while GPX4 level in group D was increased compared with group M (*p* < 0.0001). This study found that the effect of electroacupuncture therapy with distant-approximal acupoints based on the HPT axis in rats with OAT is related to the process of oxidative stress. And the main genes involved in the oxidative stress pathway were identified, which provided directions and ideas for subsequent research. But these results are only the preliminary results of transcriptomics, and relevant experiments need to be designed to further verify the mechanism of electroacupuncture therapy in rats with OAT.

## Introduction

The World Health Organization defines infertility as the inability to conceive after at least 12 months of routine, unprotected sexual intercourse [1]. As is well known, male factors account for 50% of infertility cases. Causes of male subfertility vary highly, but can be related to congenital, acquired, or idiopathic factors that impair spermatogenesis; however, the main cause is semen abnormalities [2, 3]. Male infertility can be manifested by various semen phenotypes. Oligoasthenospermia (OAT), which is defined as fewer than 16 × 10^6^ spermatozoa per milliliter of semen and less than 30% progressively moving spermatozoa, is the most common cause of male infertility [4].

OAT can be attributed to multiple pathogenic factors, which may be related to endocrine dysfunction [5]. Testicular function includes testosterone production and spermatogenesis, and both regulated by the hypothalamic-pituitary–testicular (HPT) axis [6]. Disturbance of this carefully regulated hormonal axis has caused many of the endocrine-based etiologies of oligozoospermia. In particular, any inhibition of gonadotropin secretion will have the downstream effect that disrupt testosterone biosynthesis and spermatogenesis in the testis [7].

Clinical treatments of OAT often include the use of hormones, antioxidants, and vitamins in combination with assisted reproductive technology. Traditional Chinese Medicine (TCM) often uses drugs with the effect of nourishing the kidney and nourishing the essence for treatment. However, these strategies are associated with some poor outcomes and adverse reactions [8, 9]. Therefore, there is an urgent need to explore the molecular mechanisms of OAT and more effective intervention methods.

In recent years, the advantages of nonpharmacological strategies for OAT have gradually emerged. Some guidelines and consensus lists nonpharmacological strategies as the recommended intervention for clinical treatment of OAT, mainly divided into surgical treatment, physical therapy, and TCM treatment [10]. A systematic review and Bayesian network meta-analysis showed that non-drug treatment for OAT has good clinical efficacy. Among them, the combined efficacy and safety of warm acupuncture and electroacupuncture are better [11]. Studies have shown that electroacupuncture treatment for OAT is a microtrauma surgery with less pain and can effectively improve sperm motility. However, its efficacy has not yet been scientifically and systematically evaluated [12, 13].

With the advancement of microarray and high-throughput technology, bioinformatics analysis is being used to understand the molecular mechanisms of OAT. Genomic level abnormalities, including genes and signaling pathways associated with the OAT, are increasingly being discovered. A large amount of gene sequencing data provides convenient resources for analyzing the regulation of gene expression in OAT [14, 15]. In the current manuscript, we intervened electroacupuncture therapy at distant and proximal acupoints based on the HPT axis in rats with OAT, and explored the intervention effect using transcriptomic analysis techniques.

## Materials and Methods

### Ethical Statement

All experimental procedures involved in this study were approved by the Experimental Animal Welfare Ethics Committee of the Experimental Animal Center of Ningxia Medical University (approval number: IACUC-NYLAC-2023–023, Yinchuan, China; date: 2023–09–11). It complies with animal protection, animal welfare and ethical principles, as well as relevant regulations of China's national experimental animal welfare ethics.

### Experimental Animals and Grouping

Thirty 8-week-old SPF grade male SD rats weighing 180–220 g were selected and provided by the Experimental Animal Center of Ningxia Medical University (license number: SYXK (Ning) 2020–0001). All rats were kept in the Experimental Animal Center of Ningxia Medical University under standard laboratory conditions (12:12 h light/dark schedule, humidity of 50–60%, temperature of 22–25 ℃), provided with standard rodent food and water, and allowed to eat freely. After one week of adaptation to the environment, the rats were randomly divided into a blank group (C, 10 rats) and a molding group (20 rats) using a random number table method. The blank group was given physiological saline orally, while the molding group was given continuous gavage of adenine. 28 days later, the molding group was randomly divided into a model group (M) and a electroacupuncture therapy with distant-approximal acupoints group (D), with 10 rats in each group. The M group was not treated, while the D group was treated with electroacupuncture intervention using the method of distant-approximal acupoints selection based on the HPT axis for 28 consecutive days. After the intervention, all rats were fasted for 24 h and anesthetized with 2% pentobarbital sodium intraperitoneally. After decapitation, 4 rats were randomly selected from each group to collect one side of the epididymis for sperm quality analysis. The hypothalamus, pituitary gland, and one side of the testicular tissue were used for transcriptome analysis and subsequent validation. All other rat hypothalamus, pituitary gland, and testicular tissue were immediately frozen in liquid nitrogen and stored at −80℃ for future use in experiments [16].

### Model Preparation Method

Research has shown that high-dose adenine can affect testicular function in rats, directly leading to abnormal secretion of sex hormones, disrupting the stability of the HPT axis function, and subsequently affecting sperm quality. Therefore, adenine has become one of the drugs used to construct male animal infertility models. The C group rats were given 1 ml/100 g body weight of physiological saline orally, while the molding group was given 20 mg/100 g body weight concentration of adenine orally. During the gastric lavage process, continuous stirring is required to reduce adenine precipitation and prevent blockage of the gavage needle. The medication is administered once a day for 28 consecutive days.

### Electroacupuncture Intervention Methods

Refer to Appendix II acupuncture and moxibustion Points for Common Experimental Animals of Guo Yi's Experimental acupuncture and moxibustion, and select the acupoints in combination with the Name and Location of Common Experimental Animal Points Part 2: Rats issued by the China acupuncture and moxibustion association, and select Zhongji, Guanyuan, Zusanli and Sanyinjiao. Intervention was carried out from the first day after the model was made, and disposable sterile acupuncture and moxibustion needles were used for acupuncture. After fixing the rat, expose the acupuncture site, disinfect it, fix the skin of the acupuncture site with the left hand, quickly insert the needle into the acupoint with the right hand, and connect the electrical stimulator. Using sparse and dense waves, the electroacupuncture frequency is 2 Hz, the stimulation intensity is 1.5 mA, and the degree of slight muscle tremors in the local muscles of rats is measured. Electroacupuncture intervention is performed for 30 min each time, once a day, for 28 consecutive days.

The positioning method for the four acupoints is as follows: Guan Yuan: about 25 mm below the navel; Zhongji: below Guan Yuan, at the base of the reproductive organs, on the upper edge of the inguinal ligament; Zusanli: posterior lateral side of the knee joint, about 3 mm below the fibular head; San Yin Jiao: 10 mm above the tip of the inner ankle of the hind limb.

### Epididymal Sperm Quality Analysis

Immediately dissect the epididymis and place it in physiological saline solution containing 1 m preheated to 37℃. Then gently cut open the tissue, release the contents of the epididymis, and incubate in a 37℃ constant temperature water bath for 5 min. Filter through a 200 mesh sieve, then drop 10μL of sperm suspension into a preheated sperm counting cell. We utilized the CASA computer-assisted semen analyzer (Nanning Songjing Tianlun Biotechnology Co., Ltd., model: VICOS-SPERM) to detect the count, survival rate and motility rate of sperm, in order to determine the quality of rat epididymal sperm.

### Detection of Sex Hormone Changes

Serum samples were collected from rats in each group, and the rat enzyme-linked immunosorbent assay kit was used. The corresponding operations were carried out strictly in accordance with the instructions of the kit. Within 5 min after the reaction was terminated, the absorbance value was read at 450 nm wavelength using a microplate reader and recorded. The average absorbance values of the standard and sample were calculated, and the levels of gonadotropin-releasing hormone (GnRH), follicle-stimulating hormone (FSH), luteinizing hormone (LH), and testosterone (T) in the serum of rats in each group were calculated using a standard curve.

### RNA Extraction & Library Preparation and Sequencing

We extracted total RNA from each sample's hypothalamic, pituitary, and testicular tissues using TRizol reagent (Takara, Kyoto, Japan) according to the manufacturer's instructions. Use a nanophotometric spectrophotometer (IMPLEN, Westlake Village, USA) to test the purity of RNA samples. Select a sample with an OD value of 260/280 between 1.7 and 2.5 for further analysis. Each sample was constructed into a cDNA library from lyg total RNA using the cDNA PCR sequencing kit (SQKPCS 109). In short, reverse transcriptase is used to enrich full-length cDNA, and the identified PCR adapter is added to both ends of the first strand of cDNA. Subsequently, cDNA PCR is performed 14 rounds (with an extension time of 8 min) using LongAmp Tag DNA polymerase (Biolabs, Ipswich, MA, USA). Then, the PCR product was ligated with ONT adapter using T4 DNA ligase (Biolabs, Ipswich, MA, USA). Agencourt XP beads (Beckman Coulter, Brea, USA) were used to purify DNA. The final cDNA library was analyzed on the PromethION platform of Biomarker Technology Company (Beijing, China).

### Quantification of Gene/Transcript Expression Levels and Differential Expression Analysis

Full length reads were mapped to the reference transcriptome sequence. Reads with match quality above 5 were further used to quantify. Expression levels were estimated by reads per gene/transcript per 10,000 reads mapped. Differential expression analysis of two conditions/groups was performed using the DESeq2 R package (1.6.3). DESeq2 provide statistical routines for determining differential expression in digital gene expression data using a model based on the negative binomial distribution. The resulting *P* values were adjusted using the Benjamini and Hochberg`s approach for controlling the false discovery rate. Genes with a *P* value < 0.05 and foldchange ≥ 1.5 found by DESeq2 were assigned as differentially expressed.

### Functional Annotation and Enrichment Analysis

Overall analysis was conducted on the obtained transcriptome data, and the results of principal component analysis (PCA) showed that samples from different groups were well separated. In order to understand the differences in gene expression patterns between the M and D groups of rats, differentially expressed genes (DEGs) were subjected to cluster analysis. PCA and cluster analysis performed on normalized counts. The normalization method is Z-score, and the calculation formula is: (x-mean(x))/sd(x). And study common functions or related pathways through Gene Ontology (GO) node enrichment and KEGG pathway analysis (which is the most common gene analysis method). Both KEGG and GO terms are defined as having statistical significance at *p* < 0.05. The GO enrichment and KEGG pathway analysis were performed using BMKCloud (www.biocloud.net). GO enrichment analysis of the DEGs was implemented by the GOseq R packages based Wallenius non-central hyper-geometric distribution. We used KOBAS software to test the statistical enrichment of DEGs in KEGG pathways.

### Construction of PPI Network and Identification of Genes

Based on the results and biological significance of GO and KEGG analysis, we selected genes for further research. The sequences of the DEGs was blast (blastx) to the genome of rattus norvegicus (the protein protein interaction of which exists in the STRING database: http://string-db.org/). To get the predicted PPI of these DEGs, set the combined score > 0.4, and then import the calculated results into Cytoscape-v3.9.1 to construct a PPI network. Identify the interactions between DEGs encoded proteins. And the PPI analysis results were visualized using Cytoscape software [17]. The line connecting two points in the PPI network represents the direct functional relationship between two genes. Use the CytoHubba plugin to identify hub genes using the MCC algorithm [18].

### Detection of Oxidative Stress Related Indicators

Serum samples and HPT axis tissue homogenates were collected from each group of rats, and rat enzyme-linked immunosorbent assay kit was used. Follow the instructions of the kit strictly. The average absorbance values of standards and samples were calculated, and the levels of glutathione peroxidase 4 (GPX4), superoxide dismutase (SOD), catalase (CAT) and malondialdehyde (MDA) in serum and tissue homogenates of rats in each group were calculated using the standard curve.

### Statistical Analysis

The experimental data was analyzed using SPSS 26.0 statistical software, and the results of each group were expressed as $\overline{x}\pm \text{s }$. The comparison of sample means between multiple groups that meet the requirements of normal analysis and homogeneity of variance test is conducted using one-way ANOVA, while the comparison within two groups is conducted using t-test. Non parametric tests are used for data that do not meet the above conditions. When *p* < 0.05, the difference is statistically significant.

## Results

### Analysis of Epididymal Eperm Quality

Compared with group C, group M rats showed a decrease in sperm count (*p* < 0.001), sperm survival rate (*p* < 0.001), and sperm motility rate (*p* < 0.001). Compared with group M, group D rats showed an increase in sperm count (*p* < 0.01), sperm survival rate (*p* < 0.001), and sperm motility rate (*p* < 0.001). As shown in Table 1.

**Table 1:** Analysis of epididymal sperm quality in each group of rats $\left(\overline{x}\pm \text{s }, n=4\right)$

Group	C	M	D
count(s)	318.75 ± 13.35	202.75 ± 7.54^a^	266.00 ± 7.07^b^
q		14.150	7.526
*p*		< 0.0001	0.002
survival rate(%)	87.31 ± 1.65	52.57 ± 2.82^a^	71.41 ± 1.24^b^
q		27.20	14.73
*p*		< 0.0001	< 0.0001
motility rate(%)	88.79 ± 1.54	43.21 ± 4.56^a^	60.74 ± 4.50^b^
q		26.70	11.64
*p*		< 0.0001	< 0.0001

Compared with group C, ^a^*p* < 0.05; compared with group M, ^b^*p* < 0.05

### Changes in Sex Hormone Levels

Compared with group C, the serum levels of GnRH in the group M rats decreased (*p* < 0.001), FSH increased (*p* < 0.001), LH increased (*p* < 0.001), and T decreased (*p* < 0.001). Compared with group M, group D rats showed an increase in GnRH levels (*p* < 0.001), a decrease in FSH levels (*p* < 0.01), a decrease in LH levels (*p* < 0.001), and an increase in T levels (*p* < 0.001). As shown in Table 2.

**Table 2:** Changes in serum sex hormone levels in each group of rats $\left(\overline{x}\pm \text{s }, n=5\right)$

Group	C	M	D
GnRH (mIU/L)	18.20 ± 1.35	8.58 ± 1.43^a^	15.40 ± 1.02^b^
		15.21	10.79
		< 0.0001	< 0.0001
FSH(mIU/L)	0.90 ± 0.09	2.54 ± 0.72^a^	1.40 ± 0.40^b^
		6.771	4.683
		0.002	0.033
LH (mIU/L)	6.68 ± 0.69	16.20 ± 1.34^a^	9.47 ± 0.85^b^
		18.60	13.15
		< 0.0001	< 0.0001
T (pg/ml)	65.78 ± 5.31	21.60 ± 6.26^a^	59.87 ± 4.01^b^
		14.01	12.13
		< 0.0001	< 0.0001

Compared with group C, ^a^*p* < 0.05; compared with group M, ^b^*p* < 0.05

### Identification of DEGs

**Fig. 1:**
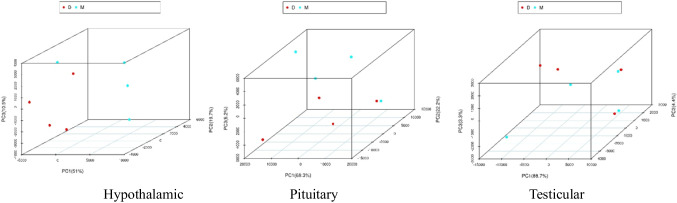
Principal Component Analysis (PCA) of DEGs of three organs along the HPT Axis in Groups D and M. the separation of rat samples from groups D and M of three different tissues was relatively significant

Perform PCA on the hypothalamus, pituitary gland, and testis of rats in groups D and M. The PCA results showed that the separation of rat samples from groups D and M of three different tissues was relatively significant, indicating that there were certain differences in the composition of gene expression between the two groups of samples from different tissues. As shown in Fig. 1.

**Fig. 2:**
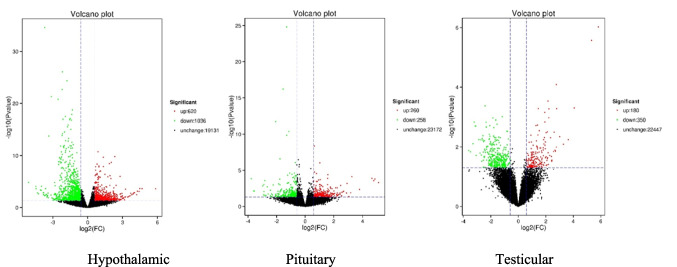
Shows the volcano plot of DEGs in three tissues along the HPT axis of rats in groups D and M. The horizontal axis represents the expression fold changes of genes in different samples, and the vertical axis represents the significance level of differences. The red dots indicate upregulated genes, while the green dots indicate downregulated genes

Genes in the hypothalamus, pituitary gland, and testis of rats in groups M and D that meet the following threshold: | Log2FC |≥ 1.5 and *P* value < 0.05 were identified as DEGs. A total of 2704 DEGs were detected in three HPT axis related tissues. Compared with group M, the volcano plot results showed that there were 620 significantly upregulated genes and 1036 significantly downregulated genes in hypothalamic tissue, 260 significantly upregulated genes and 258 significantly downregulated genes in pituitary tissue, and 180 significantly upregulated genes and 350 significantly downregulated genes in testicular tissue. As shown in Fig. 2.

**Fig. 3:**
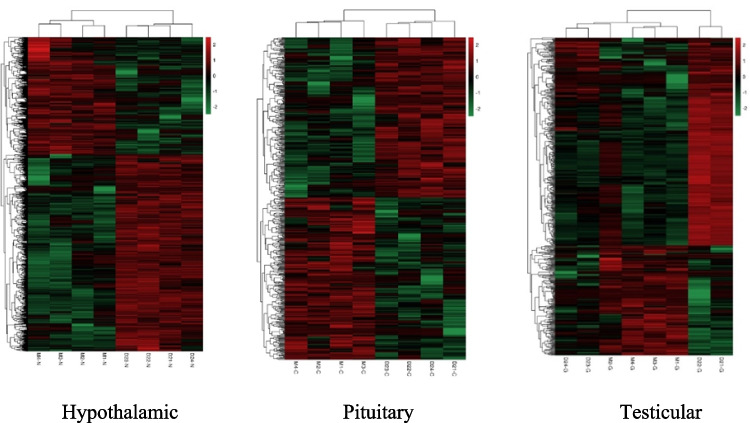
Hierarchical clustering heatmap of DEGs in three tissues of HPT axis in rats in groups D and M. The horizontal axis below represents sample names, the horizontal axis above represents sample clustering, and the vertical axis on the left represents gene clustering. The color from red to green represents gene expression from high to low

In order to understand the differences in gene expression patterns between the D and M groups of rats in the HPT axis, DEGs were subjected to cluster analysis. The results showed that the DEGs of rats in groups D and M were distributed in different sub clusters, but the four biological replicates in each group were basically distributed in the same sub cluster, which is consistent with the PCA results. There is a significant difference in distribution between Group D and Group M, which lays the foundation for our subsequent research. As shown in Fig. 3.

### Functional Enrichment Analysis of DEGs

**Fig. 4:**
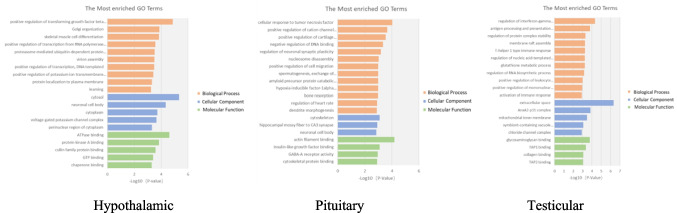
Enrichment analysis of Gene Ontology (GO) terms. GO classification DEGs were identified in hypothalamic, pituitary, and testis tissues. Orange represents Biological Process, blue represents Cellular Component, and green represents Molecular Function

To describe the functions of DEGs in the comparison between Group D and Group M of three organs, GO enrichment analysis was performed on DEGs based on the GO database. The results showed that the comparison between group D and group M with a threshold of *p* < 0.05 in hypothalamic tissue revealed 427 pathways in the biological process (BP) category, 88 pathways in the cellular component (CC) category, and 151 pathways in the molecular function (MF) category (*p* < 0.05). DEGs were mainly allocated to pathways related to cell growth, differentiation, apoptosis, and protein metabolism. The comparison between group D and group M with a threshold of *p* < 0.05 in pituitary tissue revealed 346 pathways in the BP category, 56 pathways in the CC category, and 77 pathways in the MF category (*p* < 0.05). DEGs were mainly allocated to pathways related to neural regulation and spermatogenesis. The comparison between group D and group M with a threshold of *p* < 0.05 in testicular tissue revealed 371 pathways in the BP category, 61 pathways in the CC category, and 123 pathways in the MF category (*p* < 0.05). DEGs were mainly allocated to pathways related to oxidative stress processes such as protein metabolism and glutathione metabolism. As shown in Fig. 4.

### KEGG Pathway Enriched with DEGs in HPT Axis Related Tissues

**Fig. 5:**
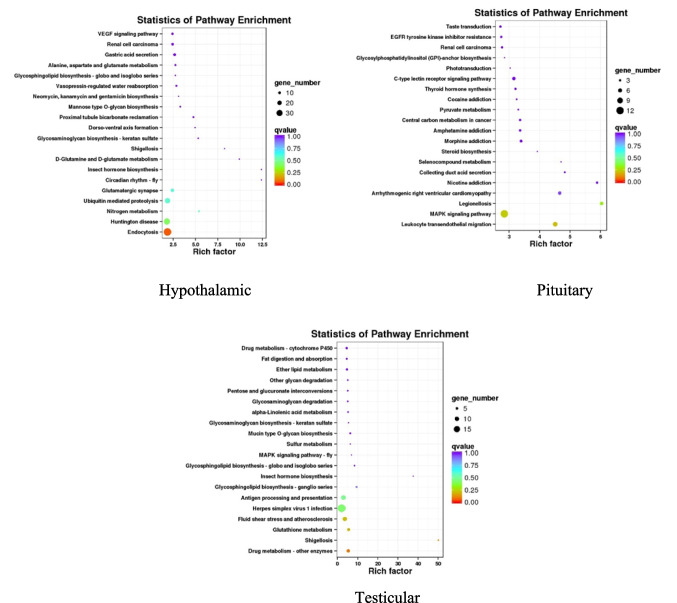
KEGG enrichment analysis of differentially expressed genes. The first 20 KEGG pathways were enriched by DEGs in the hypothalamic, pituitary, and testis tissues between group D and group M. The horizontal axis represents enrichment factors, and the vertical axis represents enrichment pathways. The size of the circle indicates the number of DEGs enriched in the pathway, and the color of the dots corresponds to different ranges of *P*-values

Compared with group M, KEGG enrichment of DEGs showed that all three tissues involved pathways related to oxidative stress. The pathway with higher enrichment of DEGs in hypothalamic tissue is the glutamatergic synaptic pathway (ko04724). In pituitary tissue, the mitogen-activated protein kinase (MAPK) signaling pathway (ko04010) is more enriched in DEGs. The pathway with higher enrichment of DEGs in testicular tissue is the glutathione metabolism pathway (ko00480). As shown in Fig. 5.

### Construction of Interactive Network for DEGs

**Fig. 6:**
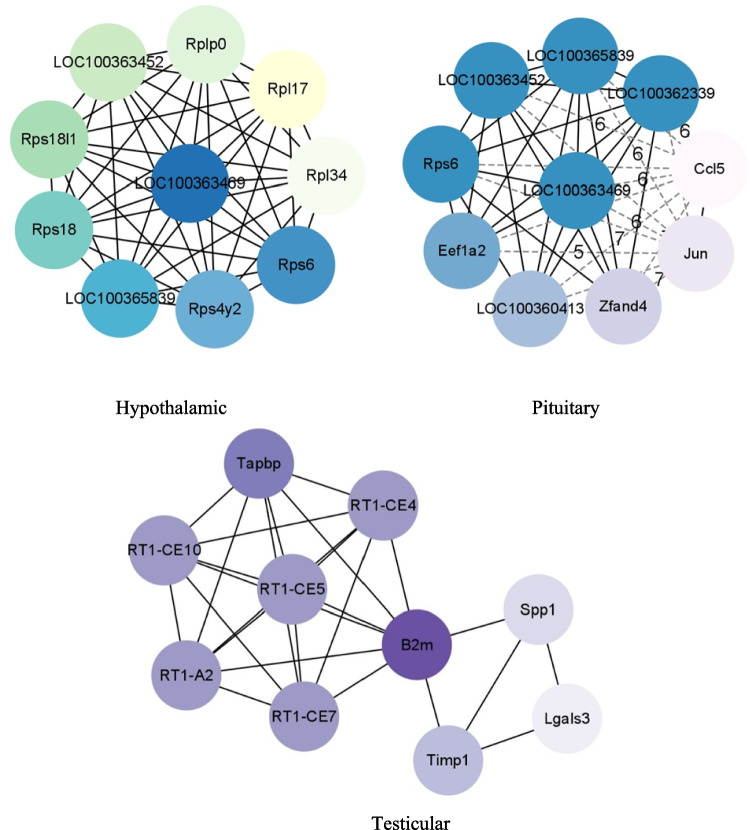
PPI network of Hub gene. The larger the connectivity, the darker the color of gene nodes

In order to reveal the gene regulatory relationship of electroacupuncture therapy with distant-approximal acupoints based on the HPT axis in rats with OAT, we constructed a PPI network by comparing the highly expressed DEGs in the D and M groups of rats in three tissues along the HPT axis. The top 10 genes with the highest connectivity to other genes were considered as hub genes. The annotations of these genes are shown in Table 3. The results showed that the Hub genes identified in the hypothalamus and pituitary tissues included *Loc100363469*, *Rps6*, *Loc100365839*, and *Loc100363452*. These genes were mainly enriched in the ribosome pathway (ko03010), which is one of the important pathways for intracellular protein synthesis and closely related to sperm production. The Hub genes identified in testicular tissue are mainly enriched in the endocytic pathway (ko04144). Sperm formation is a multi-step process characterized by the removal of excess sperm cytoplasm, the recovery of adhesion molecules through endocytosis, extensive remodeling of the cytoskeleton, and ultimately sperm separation [19]. This is closely related to the endocytic pathway. As shown in Fig. 6.

**Table 3:** Summary table of hub genes in three organs

Hypothalamic		Pituitary		Testicular	
*Loc100363469*	up	*Loc100363469*	up	*B2m*	down
*Rps6*	up	*Loc100362339*	up	*Tapbp*	down
*Rps4y2*	up	*Loc100365839*	up	*Rt1-ce4*	down
*Loc100365839*	up	*Loc100363452*	up	*Rt1-ce7*	down
*Rps18*	down	*Rps6*	up	*Rt1-ce5*	down
*Rps18l1*	up	*Eef1a2*	up	*Rt1-ce10*	down
*Loc100363452*	up	*Loc100360413*	up	*Rr1-a2*	down
*Rplp0*	down	*Zfand4*	down	*Timp1*	down
*Rpl17*	down	*Jun*	up	*Spp1*	down
*Rpl34*	down	*Ccl5*	down	*Lgals3*	down

In order to further explore the regulatory relationship between DEGs in the hypothalamus, pituitary gland, and testis, DEGs enriched in the glutamatergic synaptic pathway in hypothalamic tissue, DEGs enriched in the MAPK signaling pathway in pituitary tissue, and DEGs enriched in the glutathione metabolism pathway in testicular tissue were extracted for PPI network analysis.

**Fig. 7:**
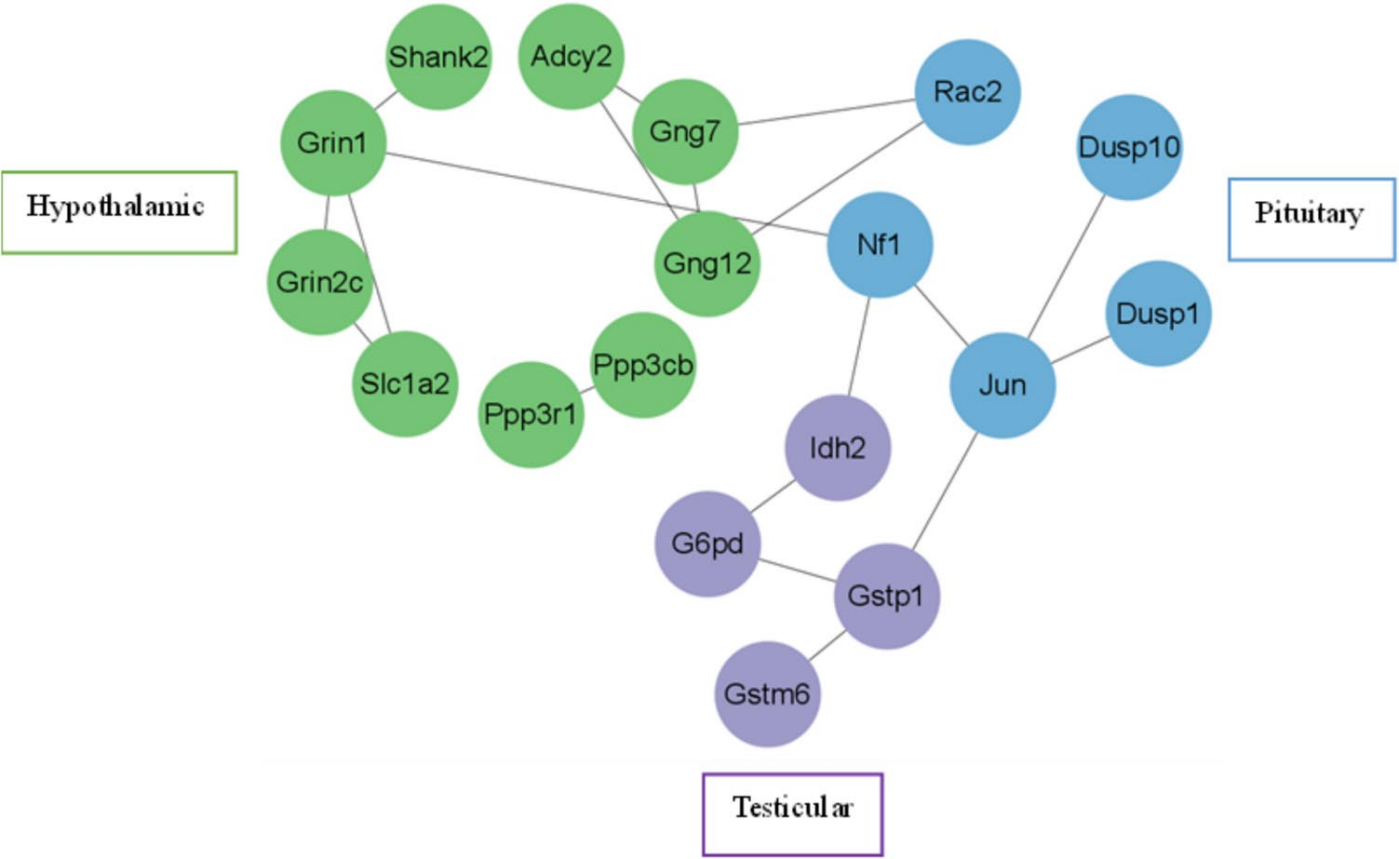
Shows the PPI network of DEGs enriched in oxidative stress pathways in three types of tissues through KEGG enrichment analysis. The green nodes represent DEGs enriched in the hypothalamus, the blue nodes represent DEGs enriched in the pituitary gland, and the purple nodes represent DEGs enriched in the testis

9, 5, and 4 DEGs were identified in the hypothalamus, pituitary gland, and testis, respectively. In this network, *Gng12*, *Grin1*, and *Gng7* are highly correlated in the hypothalamus, with *Gng12* activating PDZ protein domain binding activity and predicting involvement in the G protein coupled receptor signaling pathway; The *Grin1* gene encodes a type of glutamate receptor, which is crucial for normal signal transduction in neurons; *Gng7* is predicted to achieve G protein β subunit binding activity and participate in the regulation of G protein coupled receptor signaling pathway and adenylate cyclase activity. *Jun* and *Nf1* are highly correlated in the pituitary gland. Jun gene is not only the hub gene in the comparison of pituitary tissues between group D and group M rats, but also the DEGs enriched in the MAPK signaling pathway in pituitary tissues. It participates in multiple biological processes, including cell response to chemical stimuli, cell proliferation, and organ development. The function of *Nf1* gene is mainly to negatively regulate the Ras/MAPK signaling pathway, regulate cell growth, proliferation, and differentiation. *Gstp1* was found in testicular tissue, which is the coding gene for glutathione S-transferase pi-1 (GSTP1). The function of GSTP1 is to bind various electrophilic compounds, such as drugs, environmental toxins, oxidative chain products, etc., to glutathione and enter the next metabolic step. As shown in Fig. 7.

### Levels of Oxidative Stress Related Indicators

**Fig. 8:**
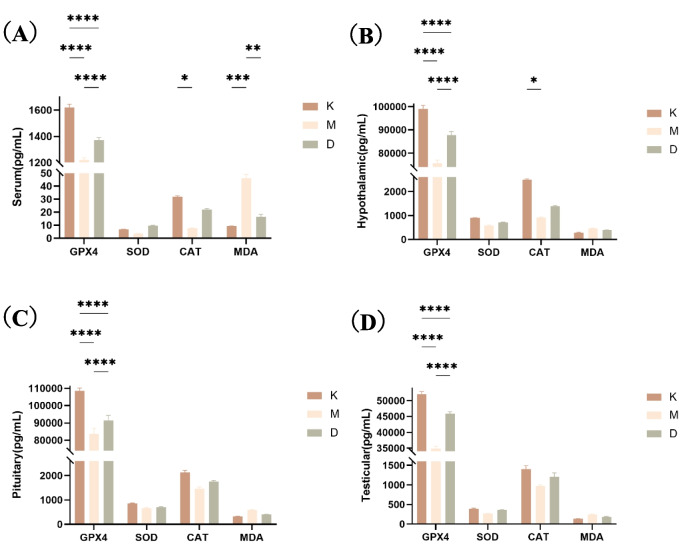
Shows the levels of oxidative stress related indicators (GPX4, SOD, CAT, MDA) in the serum (**A**) hypothalamus (**B**) pituitary gland (**C**) and testis (**D**)

No matter in serum or in three tissues of HPT axis, GPX4 level in group M was decreased compared with Group K (*p* < 0.0001), while GPX4 level in group D was increased compared with group M (*p* < 0.0001). There was no statistical difference in the level of SOD among the groups. In the serum and hypothalamic tissue, the CAT level in group M was decreased compared with that in group K (*p* < 0.05). In serum, compared with Group K, the MDA level in group M increased (*p* < 0.001), while compared with group M, the MDA level in group D decreased (*p* < 0.01). As shown in Fig. 8.

## Discussion

The results of this study showed that electroacupuncture therapy with distant-approximal acupoints based on the HPT axis could improve the number of sperm, sperm survival rate and sperm motility rate of rats with OAT by regulating sex hormone levels. In TCM, the basic pathogenesis of OAT is deficiency of kidney essence and kidney yang. Therefore, the treatment of OAT should mainly focus on tonifying kidney essence and kidney yang [20]. According to TCM, "Tiangui" is nourished by the innate essence and the acquired essence, hidden in the kidney, which not only promotes the normal development of reproductive function, but also can make up for the loss of kidney essence and repair the declining reproductive function. The function of multiple sex hormones regulated by HPT axis in regulating body growth and development is similar to that of "Tiangui" [21]. Studies have found that HPT axis dysfunction can be caused by kidney yang deficiency [22]. "Zhongji", "Guanyuan", "Zusanli" and "Sanyinjiao" are commonly used acupoints in TCM for the treatment of reproductive diseases. Their compatibility can tonify kidney essence and kidney yang, promote the arrival of "Tiangui", and embody the idea of making up for the congenital and cultivating the acquired.

Through GO enrichment analysis and KEGG pathway analysis of DEGs in group D compared with group M in three tissues of HPT axis, it was found that the effect of electroacupuncture therapy with distant-approximal acupoints based on the HPT axis in rats with OAT may be related to oxidative stress. It mainly involves the glutamatergic synaptic pathway, the MAPK signaling pathway and the glutathione metabolism pathway.

Under normal circumstances, reactive oxygen species (ROS) and antioxidants are in a dynamic equilibrium state in the body. However, in pathological conditions, a large amount of ROS is produced in the body, disrupting the dynamic balance and leading to oxidative damage [23, 24]. Due to the limited ability of sperm to resist oxidative stress damage and the limited mechanism of DNA damage monitoring and repair, sperm are vulnerable to ROS [25]. At the physiological level, ROS is crucial for sperm motility, participating in sperm maturation, hyperactivation, capacitation, acrosome response, and fertilization. However, various biological and environmental factors can cause ROS levels to rise to super physiological levels, leading to lipid peroxidation, sperm DNA breakage, and cell apoptosis, resulting in male infertility [26].

In addition, changes in oxidative stress and antioxidant system related enzyme activity may play a key role in the mechanism of spermatogenic dysfunction. Many studies have shown that testicular lactate dehydrogenase (LDH), SOD, glutathione peroxidase (GPX) activity, CAT, MDA and total antioxidant capacity (T-AOC) are indicators of oxidative stress [27, 28]. The GPX protein family catalyzes thiol redox with glutathione. Among its isozymes, GPX4 dominates in the testis and is currently considered to be essential for spermatogenesis [29]. GPX4 protects cells from oxidative stress by reducing lipid peroxide accumulation. GPX5 is only expressed in the epididymal head and may play a role in maintaining sperm DNA integrity [30]. SOD is an antioxidant metalloenzyme, which can catalyze the disproportionation of superoxide anion radicals to generate oxygen and hydrogen peroxide. CAT is an enzyme that catalyzes the decomposition of hydrogen peroxide into oxygen and water. MDA is an important bioactive molecule, which is related to oxidative stress. Human semen contains a large amount of glutathione S-transferase (GST), which can reduce the toxicity of ROS on sperm. GST plays an important role in the biotransformation and detoxification of many exogenous drugs [31]. The GST polymorphisms may have an indicative relationship between reproductive quality and sex hormone levels [32]. Oxidative stress related indicators demonstrated that OAT may be associated with reduced GPX4 levels. And electroacupuncture therapy with distant-approximal acupoints based on HPT axis can increase GPX4 levels.

It has been found that the MAPK signaling pathway is involved in the response to oxidative stress and plays a significant role in spermatogenesis, sperm meiosis, capacitation and acrosome reaction [33–36]. Several cell stimuli that induce ROS production can activate the MAPK pathway in a variety of cell types. In addition, direct exposure of cells to exogenous H_2_O_2_ would mimic oxidative stress, leading to the activation of MAPK pathway [37]. Semenza's study showed that hypoxia inducible factor 1 (HIF-1) is an important transcription factor to maintain cellular oxygen balance during oxidative stress [38]. ROS also regulate HIF-1 and inhibit its activation by chemical antioxidants [39, 40]. The MAPK signaling pathway may play a role in ROS mediated regulation of HIF-1α [41, 42]. Tong Jing Yi Hao Formula (TJYHF) is a traditional Chinese medicine for the treatment of OAT. The study found that TJYHF had beneficial effects on the reproductive function of OAT rats by inhibiting the ROS/MAPK/HIF-1 pathway [43].

The Hub genes identified in the hypothalamus and pituitary tissues included *Loc100363469*, *Rps6*, *Loc100365839*, and *Loc100363452*. These genes were closely related to intracellular protein synthesis and sperm production. The Hub genes identified in testicular tissue are mainly enriched in the endocytic pathway and related to the process of spermatogenesis. The *Jun* gene is not only the hub gene in the comparison of pituitary tissues between group D and group M rats, but also the DEGs enriched in the MAPK signaling pathway in pituitary tissues. It may be the key target of electroacupuncture therapy with distant-approximal acupoints based on the HPT axis in rats with OAT. In addition, *gng12*, *Grin1*, and *Gng7* enriched in hypothalamus and *Gstp1* enriched in testicular tissue all play a key role in the process of electroacupuncture intervention. In a similar study using a varicocele model, bioinformatics analysis revealed that the cell division cycle pathway is upregulated, while the ribosome pathway is downregulated compared to the control group [44].

This study found that the effects of electroacupuncture therapy with distant-approximal acupoints in rats with OAT is related to the process of oxidative stress, and the related pathways involved include the glutamatergic synaptic pathway, the MAPK signaling pathway and the glutathione metabolism pathway. And the main genes involved in the oxidative stress pathway were identified, which provided directions and ideas for subsequent research. This study only verified the related indicators of oxidative stress. In future studies, we can dynamically monitor the changes of oxidative stress indicators in rats after electroacupuncture intervention, and relevant experiments need to be designed to further verify the mechanism of electroacupuncture therapy with distant-approximal acupoints based on the HPT axis in rats with OAT.

## Data Availability

The datasets supporting the conclusions of this article are included within the article.
